# Integrated traditional herbal medicine in the treatment of gastrointestinal disorder: the pattern of use and the knowledge of safety among the Eastern Region Saudi population

**DOI:** 10.1186/s12906-023-04197-0

**Published:** 2023-10-23

**Authors:** Dalia Ahmed Elmaghraby, Ghufran Adnan Alsalman, Layla Hassan Alawadh, Sara Abdulaziz Al-Abdulqader, Malak Mohammed Alaithan, Bayan Ibrahim Alnuwaysir

**Affiliations:** Department of Pharmacy Practice, College of Clinical Pharmacy, King Faisal University, Al Hofuf, 31982 Saudi Arabia

**Keywords:** Phytomedicine, Gastrointestinal, Complementary therapies, Health behaviors, Herb-drug interactions, Plant preparations

## Abstract

**Background:**

Herbal medicine is commonly integrated with conventional medicine in Saudi Arabia, especially for the management of digestive disorders. However, the majority of Saudis use herbal remedies without prior consultation with a physician, which raises concerns about their appropriate and safe use. The aim of this study was to assess the level of awareness among the Saudi population regarding the proper utilization and potential adverse effects of frequently used herbs for the treatment of gastrointestinal (GI) diseases.

**Methods:**

A cross-sectional survey was conducted in Saudi Arabia from January to March 2021. An electronic self-administered questionnaire was distributed.

**Results:**

A total of 543 participants from different age groups, educational levels, and cities across Saudi Arabia completed the study questionnaire. The most commonly used herbs at home by the participants were: myrrh, parsley, black seed, chamomile, mint, anise, clove, and green tea. 57.7% of the participants perceived herbs as safer than conventional medicines; 27.3% reported that using herbal remedies over conventional medicine was a family tradition, and 21.4% used herbs because they were cheaper than conventional medicines.

**Conclusion:**

Herbal remedies, including myrrh, parsley, blackseed, chamomile, mint, and anise, are commonly employed for the treatment of gastrointestinal disorders in Saudi Arabia. However, the knowledge level of participants regarding potential side effects and drug-herb interactions was found to be deficient. As such, there is a pressing need for educational campaigns and community awareness programs to elucidate the proper usage of herbal remedies and to caution against their potential adverse effects.

## Introduction

Herbal medicine is defined as the use of natural products as a source of medicine to prevent or treat medical conditions [1, 2]. People have used plants and herbal remedies for at least 60,000 years [3]. Herbs are commonly used in Saudi Arabia for a variety of indications [4–6]. One of those indications is alleviating gastrointestinal (GI) diseases [4, 5]. Different GI diseases have different symptoms [7]. GI disorders include irritable bowel syndrome, inflammatory bowel diseases, constipation, diarrhea, dyspepsia, gastritis, and gastric ulcers [8–48].

Irritable bowel syndrome (IBS) is a functional bowel disorder that is characterized by abdominal pain, bloating, and changes in stool consistency and frequency. More than 10% of the global adult population suffers from IBS [8]. Different herbal products can be used to alleviate IBS symptoms [9–21]. Chamomile, fennel oil, anise oil, and peppermint oil can significantly decrease IBS symptoms and abdominal pain [9–15]. Anise, thyme, and marjoram have an antispasmodic and relaxant effect on the intestinal smooth muscles [16–18]. Myrrh has analgesic properties and is widely used as a home remedy in Saudi Arabia for abdominal pain and inflammatory bowel disease symptoms [9–21].

Inflammatory bowel diseases (IBDs), which include ulcerative colitis and Crohn’s disease, are abnormal mucosal immune responses with changes in the composition of gut microflora [22]. Frankincense has an anti-inflammatory effect and improves ulcerative colitis symptoms [23–25]. It can also induce remission in patients with Crohn’s disease [26, 27].

Constipation is common worldwide, especially in women and the elderly [28]. Senna is a powerful stimulant laxative that is used to treat constipation [29]. Fennel can significantly increase bowel movements and treat constipation [30].

Diarrhea serves as a significant contributor to morbidity and mortality globally, affecting individuals across all age groups [31]. Diarrhea management is usually based on stimulating water absorption which may explain the benefits of using herbs in such management [32]. Black tea is the most effective antidiarrheal herb [33]. Chamomile is a digestive relaxant and has been used to treat diarrhea, significantly reducing its duration [34].

Dyspepsia is characterized by chronic upper abdominal pain or discomfort [35]. Ginger has been used for dyspepsia to enhance GI motility and accelerate gastric emptying [36, 37]. In addition, peppermint oil has an anti-spastic effect and could help in treating dyspepsia [48]. Also, myrrh can relieve dyspepsia symptoms by stimulating the peristalsis [38].

Gastritis is an inflammation of the gastric mucosa. Gastritis incidence is reduced among people who consume green tea regularly [39, 40]. Helicobacter pylori (H. pylori) infection is the major risk factor for the development of gastritis [41]. When green tea is regularly consumed, it inhibits the growth of H. pylori [42].

Gastric ulcers are a break in the mucosal lining of the stomach or duodenum that is more than 5 mm in depth to the submucosa [43]. Fenugreek has gastroprotective and antioxidant activities which play an important role as a free radical scavenger [44]. Black seeds have a gastroprotective activity too. They significantly prevent gastric ulcer formation and reduce ulcer severity and basal gastric acid secretions [45].

Some herbs, such as green tea [46] and ginger, can support weight loss, even though implementing an effective weight loss plan can be challenging [47].

The general population considers herbal remedies to be safe [49]. Deaths and hospitalization due to herbal products are rare. However, they can cause side effects and toxicities [49]. There are at least 40 reports of toxic reactions occurring due to consuming anise teas [50, 51]. Moreover, Peppermint oil causes heartburn through the relaxation of the lower esophageal sphincter. That is why it is better used in an enteric-coated formulation [48]. Some herbs are harmful during pregnancy and should not be consumed in large amounts. Fenugreek, parsley, myrrh, and marjoram stimulate the uterus, leading to a miscarriage or premature labor [52, 53]. Senna should not be used during breastfeeding because it contains anthraquinones that are excreted in breast milk, which are genotoxic [53, 54].

Herbs may interfere with the pharmacological effect of conventional medicines [55, 56]. For example, anise has antiestrogen activity in the breast, so it can interfere with the expected effect of hormonal therapy [57, 58]. Many herbs interact with warfarin, including green tea [59], myrrh [60], anise [61], chamomile [62, 63], and fenugreek [64].

While the prevalence of gastrointestinal diseases in Arab nations is notably lower than in Western countries, there is an unfortunate upward trend in the incidence of these diseases in the Middle East and Africa [65]. This increase may be attributed to various factors, including a shift towards Western lifestyle patterns, particularly in terms of dietary habits. The adoption of a Western diet, characterized by a high intake of refined grains, saturated fats, overrefined sugars, and animal protein, is believed to predispose individuals to gastrointestinal diseases. This predisposition is likely mediated by alterations in the gut microbiota [66].

The proper use of herbs is a particular concern since much of the Saudi population uses herbal remedies on their own, without proper clinical consultation [5]. In this study, we aimed to assess the extent of community awareness regarding the proper use and side effects of commonly used herbs in treating GI diseases in Saudi Arabia.

## Materials and methods

A cross-sectional survey was conducted in Saudi Arabia from January to March 2021. A self-administered questionnaire in Arabic format was distributed electronically. Because of the public-related restrictions caused by the COVID-19 pandemic, we used several types of social media, such as Twitter, Snapchat, WhatsApp, and Instagram, to distribute the questionnaire to reach our target population.

An invitation message to participate in the study was sent via social media to family members, friends, and the general community with a link to the survey in the Google form. The snowball technique was used to collect the responses, whereby one person was invited to complete the survey and share the link with his relatives and friends. The participants were informed about the study goals and a consent form was distributed with the questionnaires.

The research team established the questionnaire based on previously published questionnaires related to herbal medicine [67–70]. The survey instrument was composed of 21 items, stratified into three distinct sections. The initial section, comprising eight questions, was designed to gather demographic data of the participants, including age, gender, educational level, presence of comorbidities, and the most commonly used herbs. The subsequent section encompassed nine questions aimed at assessing the participants’ knowledge, beliefs, and attitudes pertaining to herbal medicine. The final section consisted of four multiple-choice questions intended to solicit information regarding the perceived safety of these herbs and potential herb-drug interactions. Each correct answer was scored one point for awareness items, and a total summation of the discrete scores of the different knowledge items in the different domains (role in treating GI diseases, safety, side effects, herb-drug interaction) was calculated. A participant with a score of less than 60% (7 points or less) of the maximum score ( 13 points) was considered to have poor awareness while good awareness was considered if there was a score of 60% (8 points or more) of the maximum points.

When the participants completed the questions and submitted their answers, they received an educational poster regarding herbal medicine.

Once the research tool was translated and developed by the authors, it was sent to experts at King Faisal University for them to give their opinions and suggestions about the appropriateness of the questions, and all the necessary additions or changes in the study tools were made according to the results of the review with the research team. The Cronbach’s α for the questionnaire was 0.71, confirming its internal reliability.

A pilot study was done in 10 days, involving 20 subjects, to evaluate the clarity of the questions to the target population. All the necessary changes in the study tools were made.

The criteria for inclusion in this study encompassed all adult citizens residing in Saudi Arabia. However, we excluded any questionnaires that were incomplete or were received from individuals residing outside of Saudi Arabia.

### Ethical approval

The IRB committee of King Faisal University– (KFU- REC/2021-02-16) approved the study protocol.

### Data analysis

After data were extracted, they were revised, coded, and fed into statistical software IBM SPSS version 22(SPSS, Inc. Chicago, IL). All statistical analyses were done using two-tailed tests. A p-value less than 0.05 was statistically significant. Descriptive analyses based on the frequency and percentage distribution were done for all variables, including demographic data, medical history, awareness items, and participants’ knowledge and source of information. Cross-tabulation was used to assess the distribution of participants’ knowledge levels according to their personal and medical data. Relationships were tested using the exact probability test due to small distributions.

### Sample calculation

Utilizing an online sample size calculator provided by Raosoft®, Inc., we determined the sample size under a 5% margin of error and a 95% confidence interval. The calculated desirable sample size was 341 participants. However, the actual sample size that was achieved for this study was 543 participants.

The sample size n and margin of error E are given by:

$$\begin{array}{l}{\rm{x}}\,{\rm{ = }}\,{\rm{Z}}\left( {{\rm{c/100}}} \right){\rm{2r}}\left( {{\rm{100}}\, - \,{\rm{r}}} \right)\\{\rm{n}}\,{\rm{ = }}\,{\rm{N}}\,{\rm{x/}}\left( {\left( {{\rm{N}} - {\rm{1}}} \right){\rm{E2}}\,{\rm{ + }}\,{\rm{x}}} \right)\\{\rm{E}}\,{\rm{ = }}\,{\rm{Sqrt}}\left[ {\left( {{\rm{N}}\, - \,{\rm{n}}} \right){\rm{x/n}}\left( {{\rm{N}}\, - \,{\rm{1}}} \right)} \right]\end{array}$$

where N is the population size, r is the fraction of responses., and Z(c/100) is the critical value for the confidence level c.

## Results

The study questionnaire was completed by a total of 543 participants. The age of the participants ranged from 18 to 65 years, with an average age of 41.6 ± 12.9 years. Of these participants, 291 (53.6%) were female. The majority, 89.5%, reported having an education level of university or above. Regarding chronic diseases, 367 participants, representing 67.6% of the total, reported not having any chronic conditions, while 87 (16%) had cardiac diseases and hypertension, 70 (12.9%) had diabetes mellitus, and 34 (6.3%) had bronchial asthma (Table 1).

**Table 1:** Bio-demographic data of community participants, Saudi Arabia

Bio-demographic data	No	%
**Age in years**		
*< 35*	248	45.7%
*36–55*	226	41.6%
*> 55*	69	12.7%
**Gender**		
*Male*	252	46.4%
*Female*	291	53.6%
**Education**		
*Below university*	57	10.5%
*University*	134	24.7%
*Postgraduate*	352	64.8%
**Job**		
*Healthcare provider*	77	14.2%
*Non-health care provider*	466	85.8%
**Chronic diseases**		
*None*	367	67.6%
*DM*	70	12.9%
*Cardiac diseases and HTN*	87	16.0%
*GI diseases*	67	12.3%
*Renal diseases*	21	3.9%
*Bronchial asthma*	34	6.3%
*Hepatic diseases*	7	1.3%
*Sickle cell disease*	2	0.4%
*Others*	14	2.6%

**Fig. 1:**
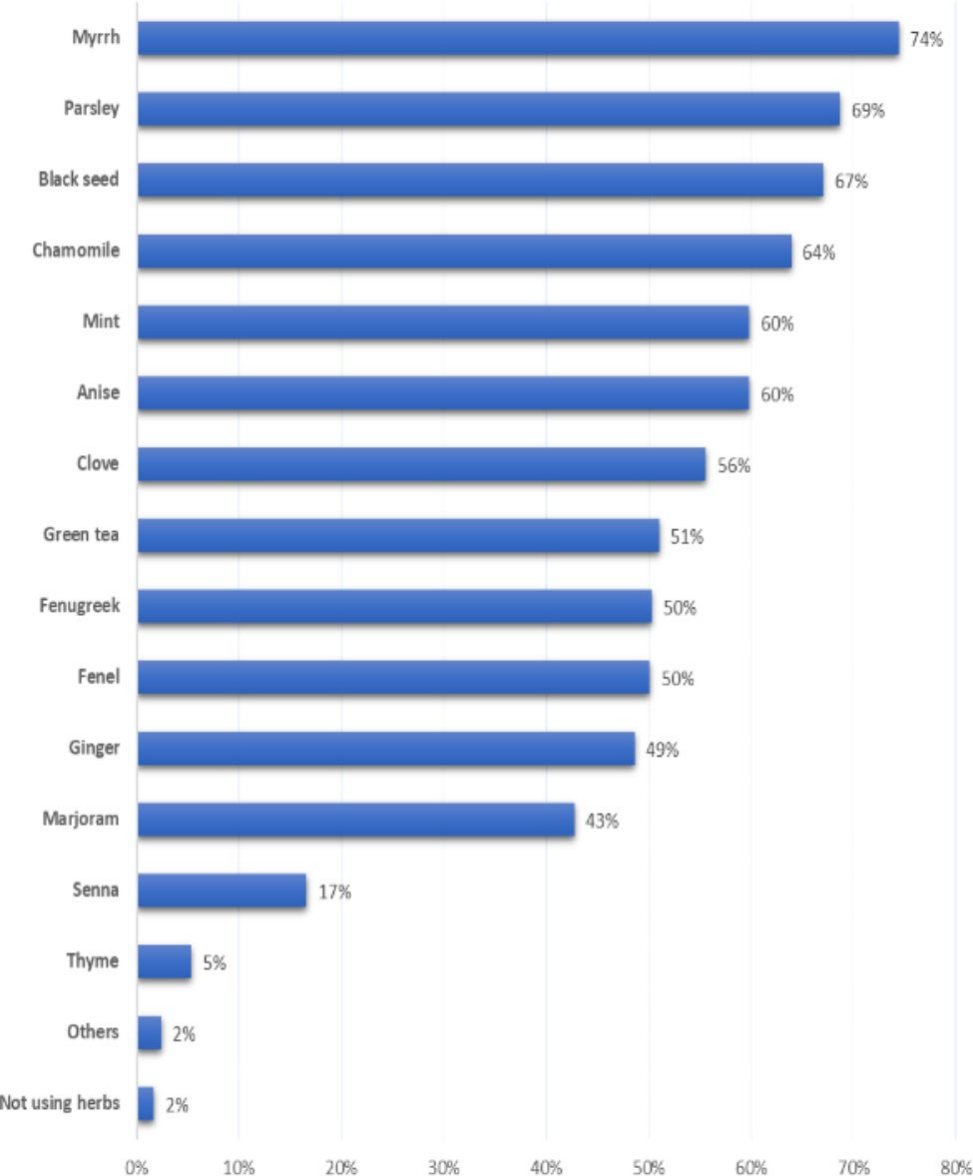
Commonly used herbs among the study participants

Myrrh was the most used herb among the study participants (74%), followed by parsley (69%), black seed (67%), chamomile (64%), mint (60%), anise (60%), clove (56%), and green tea (51%); 2% did not use herbs at home (Fig. 1).

The reasons for using herbs given by the study participants were stated as follows. Three hundred and fifty-three (65%) reported that they use herbs at home as food additives, 61.1% drank them as herbal tea and 79.9% used them as a general tonic. As for reasons for choosing herbs as a remedy, 57.7% of the respondents mentioned that herbs were safer than pharmaceutical products, 27.3% reported that it is their family tradition to use herbal remedies over pharmaceutical products, 21.4% declared that herbs were cheaper than pharmaceutical products, while 37.3% reported that they did not use herbal products as remedies (Table 2).

**Table 2:** Reasons given by the study participants for using herbs

Reasons	No	%
**The reason behind using herbs at home?**		
Food additive	353	65.0%
Herbal tea	332	61.1%
General strengthening	434	79.9%
**Reasons for choosing herbs as a remedy**		
Herbs are safer than conventional medicines.	313	57.7%
Herbs are cheaper than conventional medicines.	116	21.4%
They have a more rapid onset of action than conventional medicines.	52	9.6%
Herbs are more effective than conventional medicines.	53	9.8%
Family tradition to use herbal remedies over conventional medicines.	148	27.3%
I don’t use herbal products as herbal remedies.	202	37.3%

With reference to the participant’s knowledge of the proper use of herbs in treating GI diseases, black tea was stated by 44.9% of the participants for the treatment of diarrhea, while 12.3% knew that chamomile could be used to treat it. As for herbs that are used to treat constipation, senna was reported by 38.3% of the respondents and 28.7% reported fennel. When the participants were asked about the herbs used to treat IBS symptoms, the answers were anise 44.6%, chamomile 32.6%, and fennel 17.3%, while mint was chosen by 38.9% of the participants. Considering herbs that are used to treat dyspepsia and indigestion, mint was reported by 41.5% of the participants, while 28.4% chose ginger. Regarding herbs that are used to eradicate gastric infections, 41.7% of the participants knew about myrrh, while 18.1% chose green tea. The black seed being used to treat gastric acidity was known by 24.5% of the study respondents, and fenugreek was selected by 6.6% of them. Also, the use of fenugreek as a herb to increase body weight was known by 57.3% of the respondents. Meanwhile, 83.6% of the study respondents knew about the role of green tea in reducing body weight, and ginger was reported by 47.9% (Table 3).

**Table 3:** Participants’ knowledge regarding the proper use of herbs in treating gastrointestinal diseases

		Count	Column N %
**Which of the following herbs are used to treat Diarrhea?**	Red tea	244	44.9%
	Chamomile	67	12.3%
	Thyme	105	19.3%
	Senna	17	3.1%
	Fenugreek	31	5.7%
	Clove	11	2.0%
	Myrrh	15	2.8%
	Others	11	2.0%
	Don’t know	176	32.4%
**Which of the following herbs are used to treat constipation?**	Senna	208	38.3%
	Fennel	156	28.7%
	Thyme	106	19.5%
	Chamomile	144	26.5%
	Clove	38	7.0%
	Fenugreek	27	5.0%
	Others	16	2.9%
	Don’t know	166	30.6%
**Which of the following herbs are used to treat irritable bowel syndrome symptoms?**	Chamomile	177	32.6%
	Fennel	94	17.3%
	Anise	242	44.6%
	Mint	211	38.9%
	Thyme	89	16.4%
	Myrrh	160	29.5%
	Marjoram	162	29.8%
	Don’t know	85	15.7%
**Which of the following herbs are used to treat dyspepsia and indigestion?**	Ginger	154	28.4%
	Mint	225	41.5%
	Fenugreek	49	9.0%
	Marjoram	169	31.2%
	Fennel	102	18.8%
	Chamomile	122	22.5%
	Don’t know	112	20.7%
**Which of the following herbs are used to eradicate gastric infection?**	Myrrh	226	41.7%
	Green tea	98	18.1%
	Anise	123	22.7%
	Mint	96	17.7%
	Thyme	92	17.0%
	Fenugreek	33	6.1%
	Don’t know	152	28.0%
**Which of the following herbs are used to treat gastric acidity?**	Fenugreek	36	6.6%
	Black seed	133	24.5%
	Chamomile	99	18.2%
	Thyme	88	16.2%
	Fennel	78	14.4%
	Anise	104	19.2%
	Don’t know	240	44.2%
**Which of the following herbs are used to increase body weight?**	Fenugreek	311	57.3%
	Thyme	37	6.8%
	Anise	22	4.1%
	Parsley	46	8.5%
	Black seed	41	7.6%
	Chamomile	19	3.5%
	Don’t know	184	33.9%
**Which of the following herbs are used to reduce body weight?**	Green tea	454	83.6%
	Ginger	260	47.9%
	Parsley	116	21.4%
	Anise	58	10.7%
	Red tea	56	10.3%
	Senna	5	0.9%
	Fenugreek	32	5.9%
	Don’t know	36	6.6%

As for the safety and side effects, 25.8% of the participants knew that fenugreek should not be taken in excessive amounts by a pregnant woman, while 17.7% said myrrh and 15.8% said parsley. Considering herbs that should not be taken by a breastfeeding woman, 24.1% of the study participants chose senna. Concerning herb-drug interaction, 8% of the participants knew that chamomile could interact with medicines used to prevent blood clots, while 11.7% chose green tea, and 9.1% chose fenugreek. Considering herbs that interact with medicines used to lower blood sugar, 25.6% of the participants chose cinnamon (Table 4).

**Table 4:** Public knowledge regarding the safety and side effects of herbal medication, and herb-drug interactions

Domain	Item		No	%
**Safety and side effects**	**Which of the following herbs should not be taken by a pregnant woman in excessive amounts?**	Fenugreek	140	25.8%
		Parsley	86	15.8%
		Myrrh	96	17.7%
		Senna	190	35.0%
		Black seed	50	9.2%
		Thyme	35	6.4%
		Don’t know	236	43.5%
	**Which of the following herbs should not be taken by a breastfeeding woman?**	Senna	131	24.1%
		Fenugreek	51	9.4%
		Black seed	42	7.7%
		Mint	52	9.6%
		Thyme	30	5.5%
		Parsley	47	8.7%
		Don’t know	321	59.1%
**Herb-drug Interactions**	**Which of the following herbs interact with medicines used to prevent blood clots?**	Chamomile	43	8.0%
		Fenugreek	49	9.1%
		Green tea	63	11.7%
		Marjoram	32	5.9%
		Thyme	17	3.2%
		Anise	26	4.8%
		Don’t know	410	76.1%
	**Which of the following herbs interact with medicines used to lower blood sugar?**	Cinnamon	137	25.6%
		Fenugreek	36	6.7%
		Ginger	59	11.0%
		Red tea	40	7.5%
		Thyme	23	4.3%
		Senna	24	4.5%
		Don’t know	343	64.0%

**Fig. 2:**
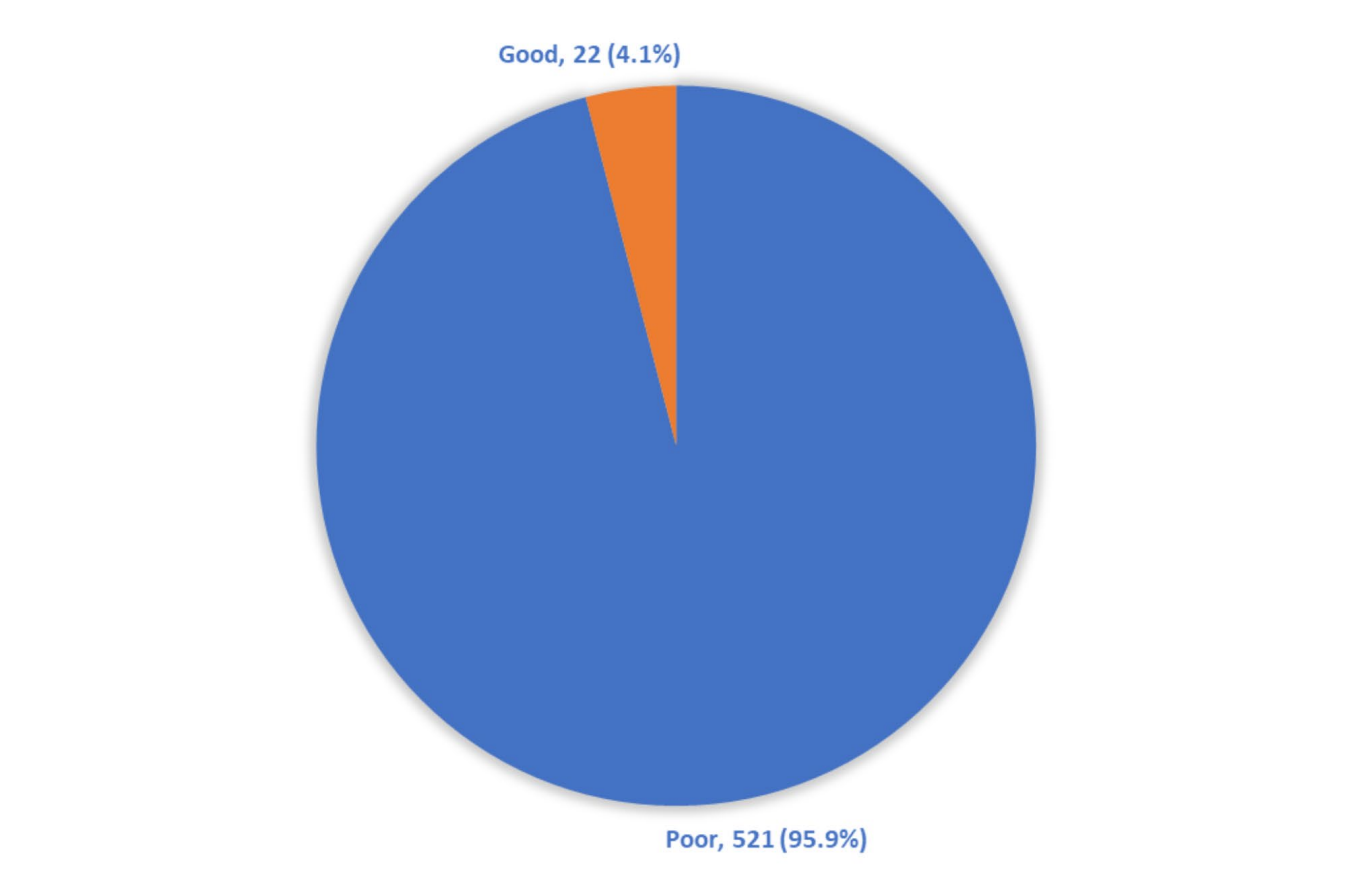
Overall participants’ knowledge level regarding the proper use of herbs in treating GI diseases

In the study, a mere 4.1% of the subjects demonstrated a comprehensive understanding of the appropriate application of herbal remedies in the management of gastrointestinal disorders (Fig. 2).

**Fig. 3:**
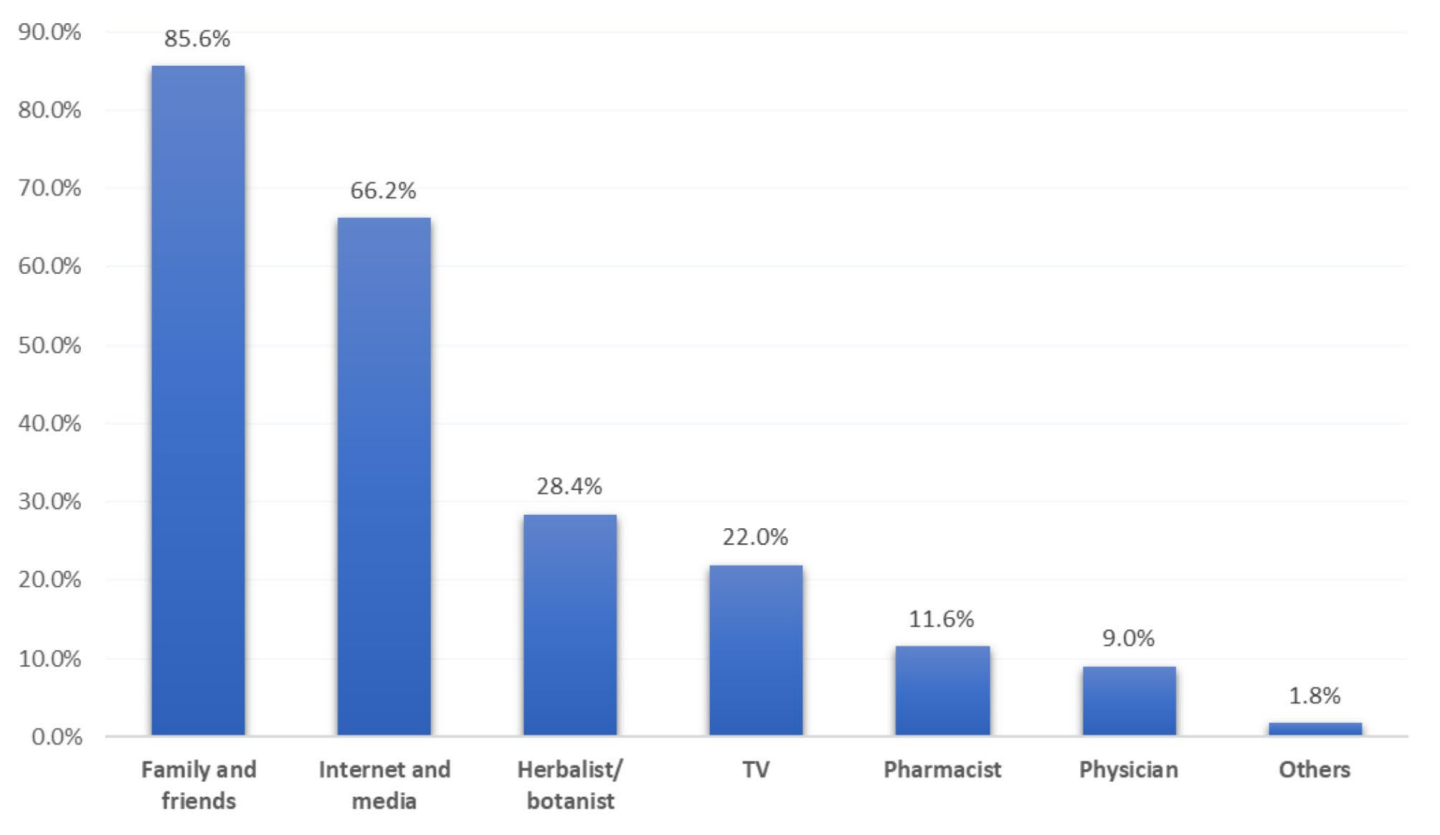
Source of information regarding safety and the use of herbs and their role in treating GI diseases

The sources of information regarding the uses and safety of herbal medication among our participants were family and friends (85.6%), followed by the internet and media (66.2%), herbalists (28.4%), and television (22%) (Fig. 3).

Of highly educated participants, 5.7% had a good knowledge level regarding herbs compared to 1.8% of those with low education; this recorded a statistical significance (P = 0.031). Besides, 11.7% of participants who were health care providers had a good knowledge level in comparison to 2.8% of others (P = 0.001). The highest knowledge level was detected among participants who got their information from a herbalist/ botanist (7.8%) or a pharmacist (7.9%) versus 3.9% of those who had received their information from family and friends (P = 0.009) (Table 5).

**Table 5:** Distribution of participants’ knowledge level regarding herbs and their uses based on their bio-demographic data

Factors	Knowledge level				p-value
	Poor		Good		
	No	%	No	%	
**Age in years**					0.900
*< 35*	239	96.4%	9	3.6%	
*36–55*	216	95.6%	10	4.4%	
*> 55*	66	95.7%	3	4.3%	
**Gender**					0.597
*Male*	243	96.4%	9	3.6%	
*Female*	278	95.5%	13	4.5%	
**Education**					0.031*
*Below university*	56	98.2%	1	1.8%	
*University*	133	99.3%	1	0.7%	
*Postgraduate*	332	94.3%	20	5.7%	
**Job**					0.001*
*Healthcare provider*	68	88.3%	9	11.7%	
*Non-health care provider*	453	97.2%	13	2.8%	
**Diseases**					0.686
*Yes*	168	95.5%	8	4.5%	
*No*	353	96.2%	14	3.8%	
**Source of information**					0.009*
*Family and friends*	446	96.1%	18	3.9%	
*Internet and media*	339	94.4%	20	5.6%	
*Television*	112	94.1%	7	5.9%	
*Herbalist/ botanist*	142	92.2%	12	7.8%	
*Pharmacist*	58	92.1%	5	7.9%	
*Physician*	47	95.9%	2	4.1%	
*Others*	10	100.0%	0	0.0%	

*P: Exact probability test*

* P < 0.05 (significant)

## Discussion

This study aimed to evaluate the extent of community awareness in Saudi Arabia concerning the correct utilization of frequently employed herbs for GI disease treatment. Additionally, it examined societal cognizance about the safety profile of herbal medications and the incidence of herb-drug interactions. According to the World Health Organization, around 80% of developing countries use traditional medicines for their primary healthcare requirements [71]. In Saudi Arabia, M. Al Akeelet et al. found that 88.7% of their study participants used herbs for therapeutic reasons [5]. In the present study, we found that 79.9% of the participants used herbal products as general tonics.

Regarding public awareness of herbs used in treating GI tract disorders, the present investigation disclosed that comprehensive awareness was notably deficient, with less than 5% of the population demonstrating adequate knowledge. Our results are consistent with another study that assessed knowledge of herbal medicine use among diabetics in Jeddah, Saudi Arabia. Most participants exhibited little knowledge [72].

The results of this study indicated that increased awareness was significantly associated with higher levels of education and employment in healthcare fields. In contrast, another study assessing the knowledge of community pharmacists regarding herbal remedies in Riyadh, Saudi Arabia, revealed that community pharmacists require additional education on herbal products due to their limited awareness. [73].

Herbal choices differ between different geographical areas. Among the study participants, the top three most commonly used herbs at home are myrrh (76%), parsley (69%), and black seed (67%). While the most commonly used herbs in Turkey are lime (80%), peppermint (68%), and rosehip (65%) [74]. whereas, the results from the 2012 American National Health Interview Survey revealed that adults with GI conditions used cranberry and echinacea to relieve GI symptoms [75].

Our study participants preferred herbal remedies over pharmaceutical products because they thought they were safer and cheaper. Meanwhile, in southwest Nigeria, people use herbs as a remedy because they think they are more effective [76].

less than half of the study participants demonstrated knowledge about herbs that can mitigate GI tract movement disorders such as diarrhea and constipation. The most effective remedies for treating diarrhea, as indicated by the responses, are black tea [33] and chamomile [34]. Only 44.9% and 12.3% chose these answers, respectively. Black tea is also often used to treat diarrhea in children in Indonesia [77]. In Saudi Arabia, Abd El-Mawla et al. reported that the prevalence of using chamomile to treat diarrhea in Taif was 13% [78]. The participants knew that senna [29] and fennel [30] were effective in treating constipation (38.8% and 28.7%, respectively). In Saudi Arabia, Abd El-Mawla et al. reported the prevalence of using senna to treat constipation at Taif as 9.4% [78]. Another study done in Riyadh reported the use of senna to treat chronic constipation as being 9.9% [79].

Only one-third of the participants correctly identified herbs that can control IBS. The best answers were chamomile, fennel, anise, mint, thyme, myrrh, and marjoram [9–21]. The percentages of participants who chose these were 32.6%, 17.3%, 44.6%, 38.9%, 16.4%, 29.5%, and 29.8%, respectively. Similarly, Abd El-Mawla et al. reported the prevalence of using anise, chamomile, and peppermint to treat IBS symptoms at Taif as being 14.74%, 10.26%, and 8.68%, respectively [78].

The participants had high awareness regarding herbs that are helpful in cases of dyspepsia, indigestion, and gastric infections. The best herbs used to treat dyspepsia and indigestion were mint and ginger [36, 37, 48]. The percentages of participants that chose these were 41.5% and 28.4%, respectively. In a prospective, double-blind, multicenter trial, 114 outpatients with chronic or recurrent functional dyspepsia were randomized and treated for four weeks with the proprietary peppermint- and caraway-oil-preparation (Menthacarin) or placebo (2 × 1 capsule/day). After two and four weeks, active treatment was superior to placebo for the relief of pain and discomfort (p < 0.001) [80]. In Turkey, Taylan Kav et al. reported that 17.2% of the respondents used Complementary and Alternative Medicine, which contains ginger, as an option to treat dyspepsia/indigestion [81].

A deficiency in knowledge was observed concerning public awareness of herbs that can be utilized for the treatment of gastric acidity. According to existing literature, fenugreek and black seed have been substantiated as effective treatments for gastric acidity [44, 45].

The highest awareness among participants was recorded for the role of herbs in managing body weight, especially related to weight reduction, which was known by more than three-quarters of the participants. Existing scientific literature substantiates the utilization of green tea and ginger for weight loss [46, 47]. The percentages of participants choosing these were 83.6% and 47.9%, respectively. Similarly, green tea and ginger are the most commonly used herbal products for weight loss among obese individuals In Dammam, Saudi Arabia [82].

A cross-sectional study conducted by Aljofan M et al. evaluated the use of herbs among pregnant women in Saudi Arabia, revealing that 33% of the participants utilized herbal medicine [70]. In relation to the use of herbs by nursing mothers, a cross-sectional study aimed to determine the prevalence, perceptions, and behaviors associated with traditional/complementary medicine use among lactating women residing in Macau. This study, conducted by Tingyun Zhen, found that 49% of breastfeeding women believed that traditional/complementary medicine was generally safe to use during lactation, while 43.6% were uncertain [83]. A systematic review of qualitative studies indicated that the use of herbal medicine by pregnant and breastfeeding women is influenced by cultural knowledge and advice from older women. [84]. Ahmad M. Eid emphasized the need for community education, particularly for pregnant and lactating women, on the correct usage of medicinal plants during pregnancy and lactation. This is crucial to minimize potential risks to fetuses and infants [85].

The current study found a low level of awareness regarding botanical medicines that should not be taken extensively by pregnant women and those that should not be taken by breastfeeding women. The herbs that should not be taken by pregnant women are fenugreek, parsley, and myrrh [52, 53]. The percentages of participants who chose these were 25.8%, 15.8%, and 17.7%, respectively. Only a quarter of our participants knew that senna should not be used in large amounts by nursing mothers ( 24.1%).

Pharmacists play a crucial role in dispensing both prescription and over-the-counter medications. As such, knowledgeable pharmacists are well-positioned to educate patients and prevent potential herb-drug interactions. Furthermore, a variety of herbal products are available for purchase in pharmacies, in different pharmaceutical forms.

In 2019, Carr, A. and Santanello, C., conducted a study of 127 community pharmacists who participated in the survey, only 34% expressed confidence in their ability to counsel patients effectively on herbal medicines. About half of the pharmacists seldom or never inquired about patients’ use of herbal medicine, and 80% rarely or never documented such use. A mere 25% of pharmacists consistently discussed side effects, and only 19% regularly addressed potential herb-drug interactions with patients using herbal medicines [86]. It is crucial to ascertain a patient’s history of herbal medicine usage prior to prescribing any medication to avoid herb-drug interaction. Herbs that interact with medicines used to prevent blood clots are chamomile, fenugreek, green tea, and anise [59–64]. The percentages of participants that chose these answers were 8%, 9.1%, 11.7%, and 4.8%, respectively. Izzo, Angelo A. et al. found, in their literature review, 43 cases and eight clinical drug interactions between cardiovascular drugs and herbal medicines, including fenugreek, green tea, and other herbs [87]. Among the participants in our study, 25.6% knew that cinnamon interacts with medicines used to lower blood sugar. A meta-analysis of 16 randomized control studies found that cinnamon consumption significantly reduced fasting blood glucose in diabetic patients. There is a need for effective education to increase awareness and ensure their safe use [88].

The strengths of our study are as follows. It is the first study to be conducted in Saudi Arabia on this topic. Furthermore, Our study encompassed participants from various cities across the eastern province of Saudi Arabia, representing a diverse range of age groups, educational levels, and genders. This diversity enhances the generalizability of our findings. On the other hand, the study’s limitation concerns the way the data were collected, which relied on self-reporting by the participants. Self-reporting is associated with an increased risk of responder and recall bias. Besides, the use of a snowball sampling technique and online survey for data collection could have missed those populations who do not use the internet.

## Conclusions

Herbal remedies, including myrrh, parsley, blackseed, chamomile, mint, and anise, are commonly employed for the treatment of gastrointestinal disorders in Saudi Arabia. However, the knowledge level of participants regarding potential side effects and drug-herb interactions was found to be deficient. As such, there is a pressing need for educational campaigns and community awareness programs to elucidate the proper usage of herbal remedies and to caution against their potential adverse effects.

## Data Availability

All data generated or analysed during this study are included in this published article. Any additional data are available upon request to the corresponding author.

## List of Abbreviations

GI: Gastrointestinal
IBS: Irritable bowel syndrome
IBDs: Inflammatory bowel diseases
H. pylori: Helicobacter pylori
